# Prevalence and Types of Child Maltreatment, and Its Impact on BMI, Smoking, and Academic Performance in Riyadh, Saudi Arabia

**DOI:** 10.7759/cureus.77782

**Published:** 2025-01-21

**Authors:** Moh’d N AlDosari, Mouath A Alturaymi, Majed Bin Dayel, Ahmed A Sharahili, Abdullah Alhejji, Abdulaziz A Alnasser

**Affiliations:** 1 Family and Community Medicine, King Saud Bin Abdulaziz University for Health Sciences, Riyadh, SAU; 2 Psychiatry, King Saud University Medical City, Riyadh, SAU; 3 College of Medicine, King Saud Bin Abdulaziz University for Health Sciences, Riyadh, SAU; 4 Family Medicine, King Saud Medical City, Riyadh, SAU; 5 Psychiatry, King Salman Hospital, Riyadh, SAU

**Keywords:** child, child maltreatment, maltreatment impact, mental abuse, saudi arabia

## Abstract

Background

Child maltreatment is behavior towards a child that deviates from the norm of conduct and carries a significant risk of physical or mental harm.All types of child abuse are considered to be child maltreatment. There are four commonly recognized types of maltreatment: physical abuse, sexual abuse, emotional abuse (psychological abuse), and neglect, with physical abuse being the most common form of child maltreatment in boys. The causes of child maltreatment are diverse and not yet fully understood. Abuse and neglect are often associated with physical injury, delayed growth and development, and mental health problems. Diagnosis is based on medical history, physical examination, and sometimes laboratory investigations and diagnostic imaging. We aim to identify the prevalence and types of child maltreatment and its impact on BMI, smoking, and academic performance.

Methods

This cross-sectional study used a self-administered questionnaire and was conducted in Saudi Arabia. It included adults of both sexes, male and female, who were living in Riyadh and were willing to participate in this study. People less than 18 years of age, living outside Riyadh, who did not consent or submitted incomplete questionnaires were excluded.

Results

A total of 385 people from Riyadh, Saudi Arabia, were included in our study. The prevalence of physical abuse was 192 (53%), 133 (37.6%)for mental abuse, 115 (11.8%)for child neglect, and 42 (32.5%)for sexual abuse. Mental abuse did not significantly impact smoking, high school academic performance, university Cumulative Grade Point Average (CGPA), or BMI. Physical abuse significantly impacted high school academic performance, but did not significantly impact smoking, university CGPA, or BMI. Sexual abuse significantly impacted high school academic performance, but did not significantly impact smoking, university CGPA, or BMI. Child neglect significantly impacted high school academic performance, but did not significantly impact smoking, university CGPA, or BMI. These results highlight the high prevalence of different forms of abuse in Riyadh and their varied impact on different aspects of individuals' lives.

Conclusion

There is a high incidence of child abuse in Riyadh. Apart from mental abuse, other types of child abusehad a substantial detrimental impact on students' academic performancein high school.

## Introduction

Child maltreatment is behavior towards a child under the age of 18 that deviates from the norm of conduct and carries a significant risk of physical or mental harm [1,2]. All types of child abuse are considered to be child maltreatment, there are four commonly recognized types of maltreatment which are physical abuse, sexual abuse, emotional abuse (psychological abuse), and neglect; with physical abuse being the most common form of child maltreatment in boys [1,3]. The causes of child maltreatment are diverse and not yet fully understood. Diagnosing child maltreatment can be challenging and is usually based on medical history, physical examination, laboratory investigations, and diagnostic imaging. Nonspecific injuries, hiding the abuse by maltreating families, and the absence of witnesses can make diagnosing and early recognizing of child abuse and neglect (CAN) difficult. Abuse and neglect are often associated with physical injury, delayed growth and development, and mental health problems [1,4,5]. Therefore, early intervention is crucial and may include documenting and treating injuries and urgent physical and mental conditions. It is also mandatory to report to appropriate government agencies, and sometimes hospitalization and/or foster care are needed to ensure the safety of the child [1].

According to WHO, around 400 million children, or 60% of those under five years old, experience physical and psychological abuse from their parents and caregivers on a regular basis. Furthermore, one in five women and one in 13 men say they have been sexually abused as children aged 0-17 years [6]. Furthermore, one in five women and one in 13 men say they have been sexually abused as children aged 0-17 years. Child abuse leads to lifelong disabilities in physical and mental health and social and occupational consequences, which can ultimately slow the economic and social development of the country. Unfortunately, violence is passed down from generation to generation, as abused children are more likely to abuse others as adults. Thus, it is important to break this cycle of violence and extensive research and studies in this field are important and needed.

A study was conducted in Al-Kharj City's schools using theInternational Society for the Prevention of Child Abuse and Neglect (ISPCAN)Child Abuse Screening Tool [7]. The study included a total of 2043 students(mean age, 16.6 years; female sex, 58%), and the incidence of psychological abuse, physical abuse, exposure to violence, neglect, and sexual abuse were 74.9%, 57.5%, 50.7%, 50.2%, and 14.0%, respectively [7]. In terms of the impact on body mass index (BMI), a meta-analysis of 41 studies found that there was an association between childhood maltreatment and obesity and maltreated individuals were more likely to be obese (odds ratio (OR)=1.36; 95%confidence interval (CI)=1.26-1.47) [8].­­

A study conducted by Altamimi et al. in intermediate and secondary schools in Riyadh, Saudi Arabia, in 2014, showed that poor performance was more likely among students who were psychologically abused versus those who were not (21.0% vs. 10.1%;p<0.01), those who were physically abused versus those who were not (18.9% vs. 9.3%;p<0.01), and those who were subject to multiple forms of abuse versus those who were not (23.4% vs. 9.7%;p<0.01) [9]. Furthermore, there appears to be an association between child maltreatment and smoking as a study from the United States indicates that a total of 81% (n = 422) of participants had experienced one or more forms of maltreatment and 16% (n = 86) reported cigarette use in the previous 30 days [10]. A study conducted in Riyadh showed that sexual assault is the most prevalent type of child maltreatment with increasing prevalence between 11-15 of age [11].

Awareness of child maltreatment needs to be evaluated in Saudi Arabia. A study revealed that the majority of Saudi school professionals have a low intermediate level of awareness about child maltreatment [12]. In addition, the prevalence of all types of child maltreatment is increased when the abused is living with the mother/father only [13]. A study done in Ecuador showed that 69.6% of the participants experienced child maltreatment during their childhood with physical abuse being the most prevalent [14]. Furthermore, a study conducted in Germany showed that physical abuse and neglect are the most commonly reported cases of child maltreatment with girls being abused more than boys [15].

Since child abuse or maltreatment has social and occupational consequences that can ultimately slow the economic and social development of the country, measuring these consequences is important to understand their magnitude and their long-term effects. Upon reviewing the literature, we did not find recent studies in Riyadh, Saudi Arabia regarding the prevalence and types of child maltreatment, and its impact on obesity BMI, smoking, and academic performance. Thus, the aim of this study was to identify the prevalence and the type of child maltreatment in Riyadh, Saudi Arabia, to find out if there is a correlation between maltreated individuals and smoking, and to assess the impact of child maltreatment on BMI and academic performance.

## Materials and methods

This was a survey that employed a retrospective cross-sectional design. Data were collected at a single point in time using a self-administered questionnaire that retrospectively addressed the past experiences of the participants from Riyadh, Saudi Arabia. Participants were recruited from various locations within Riyadh. Informed consent was obtained from all participants before they completed the questionnaire. The institutional review board of King Abdullah International Medical Research Center (KAIMRC) approved the study (approval number: NRC22R/451/09). Confidentiality and anonymity of the participants were maintained throughout the study.

Inclusion and exclusion criteria

Participants were selected based on specific inclusion criteria: adults of both genders, residing in Riyadh, and willing to participate in the study. The exclusion criteria included individuals younger than 18 years of age, non-residents of Riyadh, those who refused to participate, and incomplete questionnaires.

Sample size

The sample size was determined using Raosoft sample size calculator (Raosoft, Inc., Seattle, Washington). Parameters set for this calculation included a margin of error of 5%, a CI of 95%, a population size of approximately 4.5 million, and a response distribution of 50%. Based on these parameters, the required sample size was 385 participants. A non-probability convenience sampling technique was employed.

Data collection

Data were collected using a self-administered questionnaire, divided into two sections. The first section gathered demographic and general information, including age, gender, height, weight, smoking status, marital status, educational attainment, high school grade percentage, university Cumulative Grade Point Average (CGPA), working status, and monthly income. The second section utilized the Arabic version of the ICAST-R [16], which included 12 questions, with three questions addressing each type of child maltreatment: physical abuse, sexual abuse, emotional abuse, and neglect. The self-administered questionnaires were distributed to individuals who met the inclusion criteria and consented to participate in the study. Participants were asked to complete them on the spot or return them within a specified period. Completed questionnaires were collected and reviewed for completeness, with incomplete questionnaires excluded from the analysis.

Data analysis

Data were initially organized and reviewed using Microsoft Excel (Microsoft Corporation, Redmond, Washington, United States). Further statistical analysis was conducted using IBM SPSS Statistics for Windows, Version 27.0.1 (Released 2020; IBM Corp., Armonk, New York, United States). Descriptive statistics were used to summarize the data, and inferential statistics were applied to identify significant patterns and correlations among the variables. The primary focus was on understanding the prevalence and types of child maltreatment and exploring the potential impacts of these experiences on the participants' current demographic and socioeconomic status. The sociodemographic characteristics frequency, percentage, mean, and standard deviation (SD) of participants who were maltreated as children in Riyadh, Saudi Arabia, were calculated. The chi-squared test, bivariate correlation with Spearmen Rho Test, and Man-Whitney U Test for non-parametric data were applied to find the association between different variables and maltreated children in Riyadh. Descriptive statistics of age and gender were presented as frequencies and percentages. A statistically significant test value was defined as a p-value < 0.05.

## Results

The study on child maltreatment in Riyadh explored the prevalence and impact of different types of abuse, physical, mental, sexual, and neglect, among 385 participants. Demographic data is further illustrated in Table 1. The prevalence rates in Riyadh were found to be quite high: 53% (n= 192) for physical abuse, 37.6% (n=133) for mental abuse, 11.8% (n=115) for child neglect, and 32.5% (n=42) for sexual abuse as illustrated in Table 2.

**Table 1:** Sociodemographic parameters (N=385)

Parameters		Frequency	Percentage
Gender	Male	279	72.5%
	Female	106	27.5%
City	Riyadh	353	91.7%
Smoking (n=98)	Before 18 years of age	48	12.5%
	After 18 years of age	50	13%
Marital Status	Single	320	83.1%
	Married	57	14.8%
	Divorced	6	1.6%
	Widowed	2	.5%
Work	Full-time with pay	111	28.8%
	Work without pay	9	2.3%
	Don’t work	265	68.8%
Monthly Income of People (Saudi Riyal)	< 5000	277	71.9%
	5000-10,000	52	13.5%
	10,000-15,000	25	6.5%
	>15,000	31	8.1%
Educational Status	Illiterate	2	.5%
	Read and write	4	1.0%
	Middle school	14	3.6%%
	High school	129	33.5%
	Diploma/Vocational	89	23.1%
	University/Postgraduate	147	38.2%

**Table 2:** Frequency and prevalence of different types of maltreatment reported by the participants (N=353)

Different Types of Maltreatment	Frequency	Percentage
Physical Abuse	192	53.3%
Mental Abuse	133	37.6%
Sexual Abuse	115	32.5%
Child Neglect	42	11.8%

The study also examined how these types of abuse influenced various factors such as smoking behavior, gender differences, BMI as illustrated in Table 3, educational performance in high school as illustrated in Table 4, and university CGPA as illustrated in Table 5.

**Table 3:** Mean of BMI and its comparison among different types of maltreatment Postitive value in mean difference show increase in mean value in abused individuals

BMI		Mean	Standard Deviation	Difference in Mean	p-value
Overall		25.13	7.096		
Physical Abuse	Yes	25.59	7.931	.892	0.531
	No	24.70	5.716		
Mental Abuse	Yes	25.67	8.150	1.258	0.382
	No	24.41	6.143		
Sexual Abuse	Yes	25.91	8.843	1.258	0.526
	No	24.29	5.658		
Child Negligence	Yes	26.34	12.373	.230	0.841
	No	26.11	7.089		

**Table 4:** Mean of high school educational performance and its comparison among different types of maltreatment Negative value in mean difference show decrease in mean value in abused individuals *significant

High School Educational Performance		Mean (%)	StandardDeviation	Difference in Mean	p-value
Overall		93.43	7.096		
Physical Abuse	Yes	92.70	7.670	-1.662	0.007^*^
	No	94.36	7.139		
Mental Abuse	Yes	92.99	8.058	-1.794	0.296
	No	94.78	5.924		
Sexual Abuse	Yes	92.89	6.926	-.899	0.047*
	No	93.79	7.657		
Child Negligence	Yes	89.51	9.654	-2.929	0.031^*^
	No	92.43	8.096		

**Table 5:** Mean of university CGPA and its comparison among different types of maltreatment Positive value in mean difference show increase in mean value in abused individuals; negative value in mean difference show decrease in mean value in abused individuals *significant CGPA: cumulative grade point average

CGPA in University		Mean (%)	StandardDeviation	Difference in Mean	p-value
Overall		4.66	7.119		
Physical Abuse	Yes	4.89	8.957	.822	0.635
	No	4.07	.665		
Mental Abuse	Yes	4.13	.613	-.985	0.946
	No	5.11	9.846		
Sexual Abuse	Yes	4.04	.668	-1.320	0.701
	No	5.36	10.853		
Child Negligence	Yes	3.83	.760	-.259	0.031^*^
	No	4.09	.613		

Mental abuse

The study found no significant association between mental abuse and smoking (p=0.677), gender (p=0.083), high school educational performance (p=0.297, r=0.056), university CGPA (p=0.946, r=0.004), or BMI (p=0.382, r=-0.045). This suggests that childhood mental abuse did not have a notable impact on these parameters in adulthood.

Physical abuse

Physical abuse showed no significant effect on smoking behavior (p=0.074) or university CGPA (p=0.636, r=0.027). However, it did show a significant difference based on gender (p=0.019), indicating that one gender experienced more physical abuse. It also significantly affected high school educational performance (p=0.007, r=0.145), with those who experienced physical abuse performing worse academically. BMI was not significantly affected by physical abuse (p=0.532, r=-0.032).

Sexual abuse

Sexual abuse did not significantly impact smoking (p=0.530), gender (p=0.109), university CGPA (p=0.702, r=0.022), or BMI (p=0.527, r=-0.032). However, it significantly affected high school educational performance (p=0.047, r=0.107), with those who experienced sexual abuse having lower academic performance.

Child neglect

Child neglect showed no significant association with smoking (p=0.439) or university CGPA (p=0.155, r=0.082). However, it did show significant differences based on gender, with females experiencing more neglect than males (p=0.005). Child neglect also significantly affected high school performance (p=0.031, r=0.115) as shown in Table 4, with those neglected performing worse academically. Although BMI was generally not significantly impacted by neglect (p=0.842, r=0.010) as shown in Table 3, individuals without a history of neglect tended to have lower BMI, which was notable (p<0.05).

## Discussion

Our study discovered that half of the participants had an experience with physical abuse and others reported mental abuse, sexual abuse, and neglect. To be specific, the study revealed a prevalence of 53% (n=192) for physical abuse, 37.6% (n=133) for mental abuse, 32.5%(n=115) for sexual abuse, and 11.8% (n=42) for child neglect. On the other hand, according to asystematic review, in child maltreatment globally, the prevalence of physical abuse is 22.6%, emotional abuse is 36.3%, sexual abuse is 18%, and child neglect is 16.3%[17]. In Saudi Arabia,one study found a total of 188 referrals to the CAN team, of which 133 (70.7%) were confirmed as CAN cases; the number of CAN cases referred to the team increased significantly over the three time periods, from 6.4 cases per year in the first period to 61.5 cases per year in the third period [18]. The most common form of abuse shifted from physical abuse in the earlier periods to neglect in the later period. These findings show that Riyadh has a notably higher prevalence of child maltreatment in the global context, especially in physical and sexual abuse, while in the local context, the results are somewhat comparable.

Mental abuse

No significantconnection was found between childhood mental abuse and smoking, high school educational performance, university CGPA, or BMI in adulthood.This is possibly due to the complexity ofmental abuse and its long-term psychological impacts, which may not beeasilycaptured by academic performance orevenphysical health metrics.Also, it is important to considerthe possibility of underreporting. Finally, a study revealedthat childhood adversities involving maladaptive family functioning (e.g., parental mental illness, substance use disorder, family violence, physical and sexual abuse, and neglect) were significantly but modestly associated with the persistence of mood, substance abuse, and anxiety disorders, with subadditive effects observed for multiple exposures [19].

Impact of physical abuse

There was a notable negative effect of physical abuse on high school education,which indicates that the issuecan hinder academic performance during such important years, leading to long-term implications forcareer opportunities and socioeconomic status.However, the lack of a significant link between physical abuse with university CGPA might hint toward some form of recovery or correction mechanisms during later academic stages, or it could show the significant challenges for those who reach university.In Jeddah, Saudi Arabia, a study showed thatphysically abused students were more likely to have a GPA below 85% compared to non-abused students[20].The present study corroborates the negative impact on academic performance.

Impact of sexual abuse

Lower academic performance in high school was noted due to the significant effect of sexual abusewhichunderscores the severe impact of such trauma on academic outcomes.Additionally,psychological distress, concentration difficulties, or absenteeism resulting from the abusecan be reasons for lower high school performance among victims. In terms of sexual abuse, the associations with smoking, university CGPA, or BMI were absent which might reflect the deeply personal nature of sexual abuse experiences, which can affect individuals in diverse ways that may not be easily measurable.One study reported that individuals who experienced sexual abuse had poorer educational outcomes and higher rates of smoking and substance abuse in adulthood[21].Ourstudy aligns with the educational impact but not with smoking behavior.

Impact of child neglect

We hadnotable findings that female respondents had faced more neglect than male respondents, highlighting the significance ofthisissue. An explanation is that itmightbe rooted in cultural or societal norms.Thus, it is necessary to advocate for focused interventions to alleviate and prevent neglect, especially among girls. Furthermore, the detrimental effects of neglect on high school performance are consistent with the knowledge that neglect can deteriorate cognitive and emotional development, which in turn results in subpar academic achievement. Furthermore, while the association between neglect and BMI is not statistically significant overall, it may imply that people who have been neglected may have higher BMIsbecause ofcoping strategies like overeating or limited access to opportunities for exercise and goodfood.Astudy in the United Kingdom found that neglected children had significantly lower academic achievement and higher BMI in adulthood[22]. This is consistent with the findings regarding academic performance but only partially aligns with the BMI results.

Recommendations

The high incidence rates of different forms of child abuse necessitate the implementation of strong laws and community-based programs that emphasize victim support, early detection, and prevention. Programs tailored to a person's gender may be required to address theabuseexperiences and vulnerabilities that each gender has. Educating parents, educators, and healthcare professionals on the warning signs and outcomes of child abuse can aid in early detection and intervention. It is imperative that educational institutionshavethe requisite resources to assist students who have been victims of abuse, guaranteeing that they have essential academic and psychological assistance. Further investigation is required to examine the long-term psychological effects of various abuse scenarios, going beyond the scope of this study's measurements.Establishing best practices for supporting victims and stopping abuse can be aided by researching the efficacy of different intervention techniques. This study emphasizes the urgent need for all-encompassing approaches to stop child abuse in Riyadh, with an emphasis on healing, assistance, and prevention to lessen the long-term effects on those impacted.

Strength and limitations

Being the first of its kind in Riyadh, our study highlights a highincidence of different types of child maltreatment and its impact among our studied population projecting the need for awareness and training programs for caregivers, teachers, and healthcare providers, further enforcement of child protection laws by policymakers, and support systems and counseling services implementations in academic institutions. However, there are severallimitations of the study. First, the only method of data collection was questionnaire distribution. Second, as per the inclusion/exclusion criteria, 32 patients living outside the capital had to be excluded. Also, third, this study was cross-sectional which limits its generalizability.

## Conclusions

The study demonstrates the high incidence of child abuse in Riyadh. Additionally, it shows that child abuse has a substantial detrimental impact on academic performance, especially in high school. These effects may have long-term effects on the victims' socioeconomic standing and future prospects. While the effects of sexual and physical abuse were more noticeable in terms of educational outcomes, more research is needed to fully understand the psychological effects of mental abuse and neglect, especially in light of the possibility that these effects go unreported and the intricate long-term effects they can have on mental health. The results highlight the necessity of comprehensive programs, gender-specific initiatives, early detection techniques, strong legislation, and victim education and support networks in order to effectively combat child abuse in Riyadh. In addition, better prevention and support systems depend on ongoing research into the long-term psychological effects of abuse and the effectiveness of intervention techniques. Ultimately, to lessen the severe effects of child abuse in Riyadh, a comprehensive strategy centered on healing, prevention, and support is required.

## Figures and Tables

**Figure 1:**
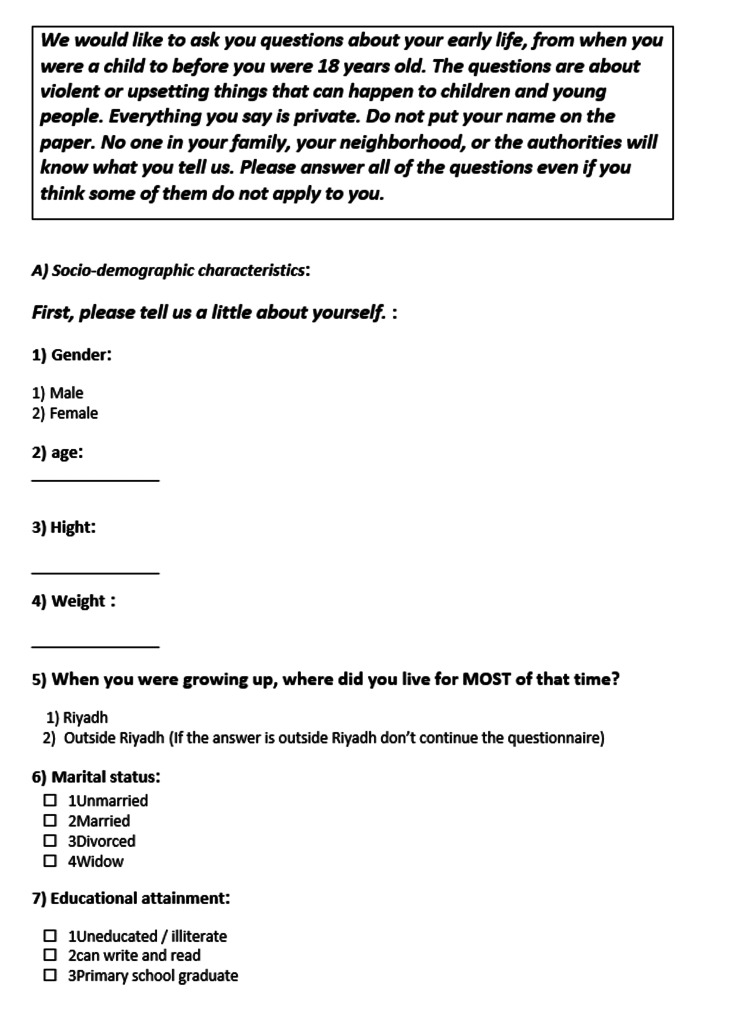
Page 1 of the questionnaire

**Figure 2:**
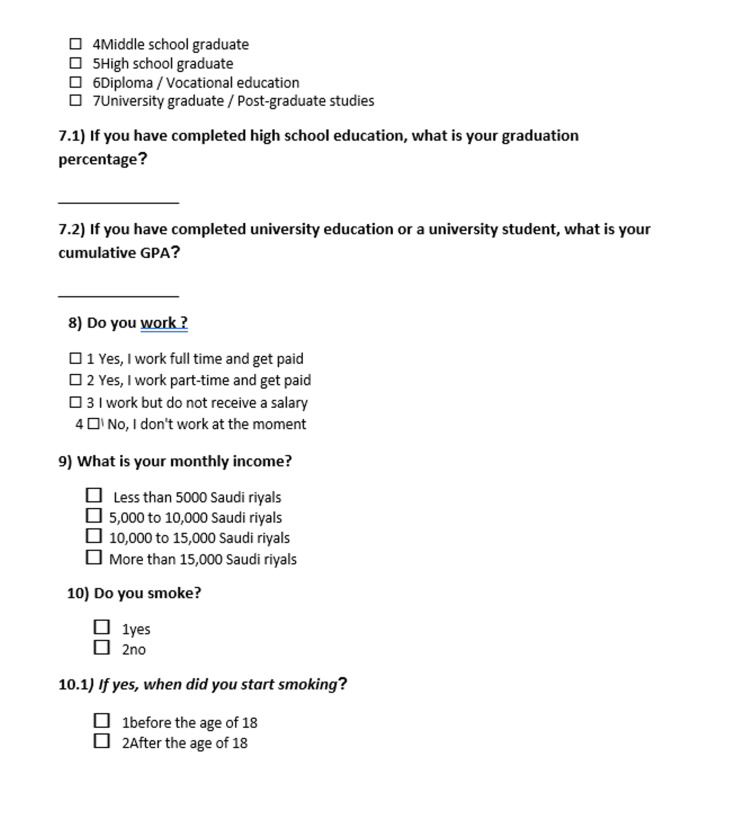
Page 2 of the questionnaire

**Figure 3:**
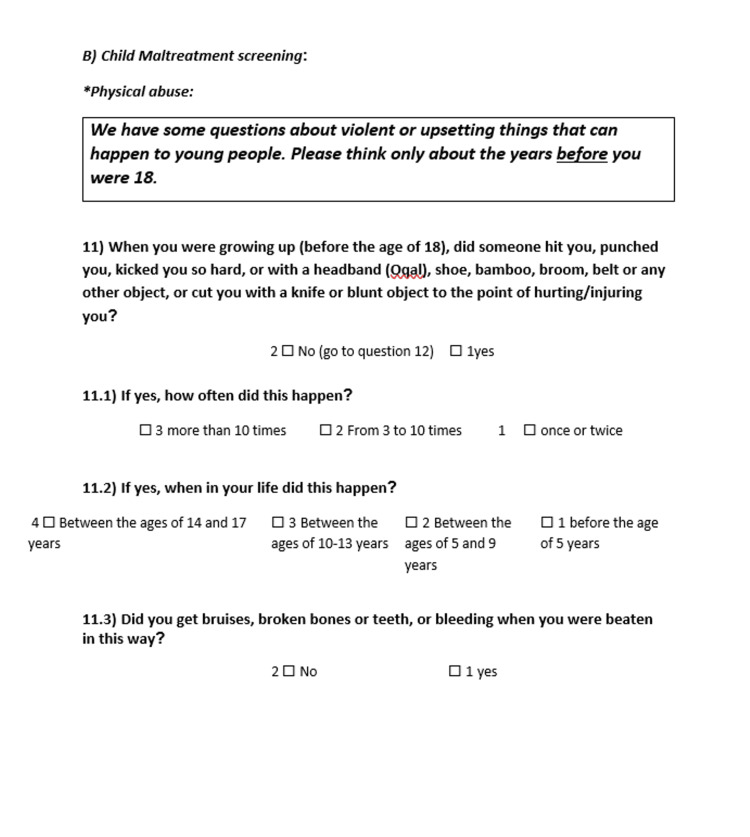
Page 3 of the questionnaire

**Figure 4:**
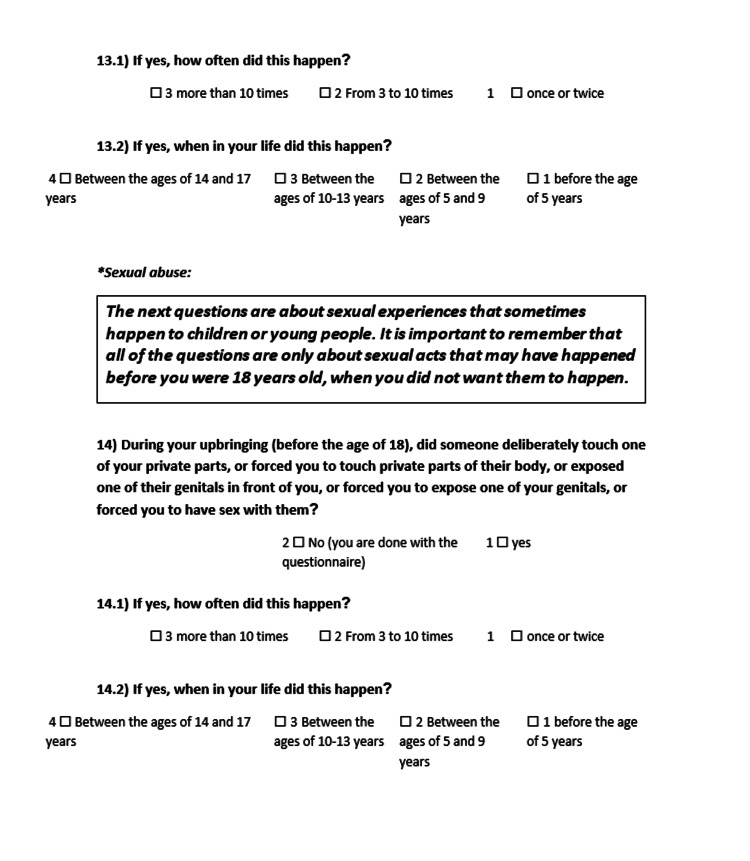
Page 5 of the questionnaire

## Appendices

English version of the questionnaire used in the study

Arabic version of thequestionnaire used in the study

نود أن نسألك عن حياتك منذ طفولتك حتى قبل بلوغ سن الثامنة عشر. الأسئلة عن العنف أو الأشياء المزعجة التي قد يتعرض لها الأطفال أو الشباب. كلاجاباتك على الأسئلة ستكون خاصة و سرية للغاية. لا تضع اسمك على الاستبيان. لا يحق لأي فرد من عائلتك، من معارفك أومن المسئولين الإطلاع على ما تدلي به. نرجو الإجابة على جميع الأسئلة حتى لو لم تنطبق عليك بعضها.

أ) الخصائص الاجتماعية والديموغرافية:

نبدأ أولاً بالتعرف عليك:

1) الجنس:

1) ذكر

2) أنثى

2) العمر:

______________

3) الطول:

______________

4) الوزن:

______________

5)خلال فترة طفولتك، أين قضيت أو عشتمعظمحياتك؟

1) الرياض

2) خارج الرياض (إذا كانت الإجابة خارج الرياض لا تكمل الاستبيان)

6) الحالة الاجتماعية:

¨1 أعزب

¨2 متزوج

¨3 مطلق/ منفصل

¨4 أرمل

7) ما هو تحصيلك العلمي؟

¨1 غير متعلم/ غيرمتعلمة/ أُمي/أُمية

¨2 أجيد القراءة والكتابة

¨3 أكملت التعليم الابتدائي بنجاح

¨4 أكملتالتعليم المتوسطبنجاح

¨5 أكملتالتعليم الثانويبنجاح

¨6 تعليم ما بعد الثانوي/ الدبلوم / التعليم المهني

¨7 حصلت على درجة التعليم الجامعي او الدراسات العليا

7.1) إذا كنت قد أكملت التعليم الثانوي، ماهي نسبتك المئوية؟

______________

7.2) إذا كنت قد أكملت التعليم الجامعي او طالب جامعي، ما هو معدلك التراكمي (GPA)؟

______________

8) هل تعمل؟

1 نعم، أعمل بدوام كامل و أتقاضى راتب

2 نعم، أعمل نصف دوام و أتقاضى راتب

3 أعمل و لكن لا أتقاضى راتب

4لا، لا أعمل حالياً

9) ما هو دخلك الشهري؟

£أقل من 5000 ريال سعودي

£5000 الى 10000 ريال سعودي

£10000 الى 15000 ريال سعودي

£اكثر من 15000 ريال سعودي

10) هل تدخن؟

£1 نعم

£2 لا

10.1) إذا كانت الإجابة نعم، متى بدأت بالتدخين؟

£1 قبل سن ال 18

£2 بعد سن ال 18

ب) تحري إساءة معاملة الأطفال:

ثانياً،لدينا بعض الأسئلة عن الأشياء العنيفة أو المزعجة التي قد يتعرض لها الأولاد و الشباب. تذكر فقط ما يتعلق بالفترةما قبل 18 سنة.نحن نتفهم أن هناك أمور سلبية في بعض هذه الأسئلة أو قد لا تنطبق عليك أو على ثقافتك، و لكن نرجو منك ملء الاستبيان بناء على تجربة نشأتك.

11) خلال نشأتك ( قبل سن 18)، هل ضربك أحدبشدةلكمك (بوكس) أو ركلك (رفسك) أو بواسطة عقال، حذاء، خيرزان، مكنسة، حزام أو أي شيء آخر أو جرحك بسكين او بآلة حادة لدرجة إيذائك/ مما أدى إلى إصابتك؟

1 نعم

2لا(اذهب لسؤال رقم12)

11.1) إذا كانت الإجابة نعم، كم مرة حصل ذلك؟

1مرة أو مرتين

2من 3 إلى 10 مرات

3أكثرمن 10 مرات

11.2) إذا كانت الإجابة نعم، في أي وقت من حياتك حدث هذا؟

1 قبل سن 5 سنوات

2 بين سن 5 و9 سنوات

3 بين سن 10-و13 سنة

4بين سن 14 و17 سنة

11.3) هل نتج عند تعرضك للضرب بهذه الطريقة لكدمات، كسر في العظام أو الأسنان أو نزيف؟

1 نعم

2لا

في بعض الأحيان قد يشعر الأطفال بالقلق أو الخوف الشديد. قد يؤدي ذلك إلى شعورهم بالحرج، الخجل أو بعدم الحب. أجب عن الأسئلة التالية التي تدور حول الأمور التي قد تكون حدثت لك قبل بلوغك سن ال18.

12) خلال نشأتك (قبل سن 18)، هل أهانك و انتقدك أحد مما أدى إلى شعورك بأنك سيء، أحمق أو عديم الفائدة، أو قال لك أحد أفرادأسرتك أو عائلتكبأنك غير محبوب أو أنك لا تستحق الحب أو بأنه يتمنى ألا تكون وُلدت أو مُت أو تعرضت شخصياً للتهديد بإيذائك أو بقتلك؟

2لا(اذهب لسؤال رقم13)

1نعم

12.1) إذا كانت الإجابة نعم، كم مرة حصل ذلك؟

1مرة أو مرتين

2من 3 إلى 10 مرات

3أكثرمن 10 مرات

12.2) إذا كانت الإجابة نعم، في أي وقت من حياتك حدث هذا؟

1 قبل سن 5 سنوات

2 بين سن 5 و9 سنوات

3 بين سن 10-و13 سنة

4بين سن 14 و17 سنة

في بعض الأحيان الآباء والأمهات أو أولياء الأمور لا يستطيعون او لا يهتمون بتقديم الرعاية أو الاهتمام الكافي للطفل. أجب عن الأسئلة التالية التي تدور حول الأمور التي قد تكون حدثت لك قبل بلوغك سن ال18.

13) خلال نشأتك (قبل سن 18)، هل سبق ان تعرض الى اصابة بسبب عدم اشراف الوالدين او احد الكبار أو لم تقدم لك الرعاية الكافية عند المرض والاصابة أو لم يتوفر لك الطعام والشراب والملابس او احد الاحتياجات الأساسية على الرغم من أنهم يستطيعون تحمل تكاليفها؟

2لا(اذهب لسؤال رقم14)

1نعم

13.1) إذا كانت الإجابة نعم، كم مرة حصل ذلك؟

1مرة أو مرتين

2من 3 إلى 10 مرات

3أكثرمن 10 مرات

13.2) إذا كانت الإجابة نعم، في أي وقت من حياتك حدث هذا؟

1 قبل سن 5 سنوات

2 بين سن 5 و9 سنوات

3 بين سن 10-و13 سنة

4بين سن 14 و17 سنة

14) خلال نشأتك (قبل سن 18)، هل تعّمّد أحد لمس إحدى الأماكن الحساسة بجسمك أو أجبرك أحد على لمس أماكن حساسة بجسمه أو كشف أحد أعضائه التناسلية أمامك أو اجبرك بكشف احد اعضائك التناسلية أو أجبرك أحد على ممارسة الجنس معه؟

2لا(اذهب لسؤال رقم)

1نعم

14.1) إذا كانت الإجابة نعم، كم مرة حصل ذلك؟

1مرة أو مرتين

2من 3 إلى 10 مرات

3أكثرمن 10 مرات

14.2) إذا كانت الإجابة نعم، في أي وقت من حياتك حدث هذا؟

1 قبل سن 5 سنوات

2 بين سن 5 و9 سنوات

3 بين سن 10-و13 سنة

4بين سن 14 و17 سنة
